# Supplementary material for: Synthetic Lethality Screening Identifies FDA-Approved Drugs that Overcome ATP7B-Mediated Tolerance of Tumor Cells to Cisplatin
Source: Cancers (Basel). 2020 Mar 6;12(3):608. doi: 10.3390/cancers12030608 (PMC7139527; doi:10.3390/cancers12030608)

Original Western blots for Figure 1C

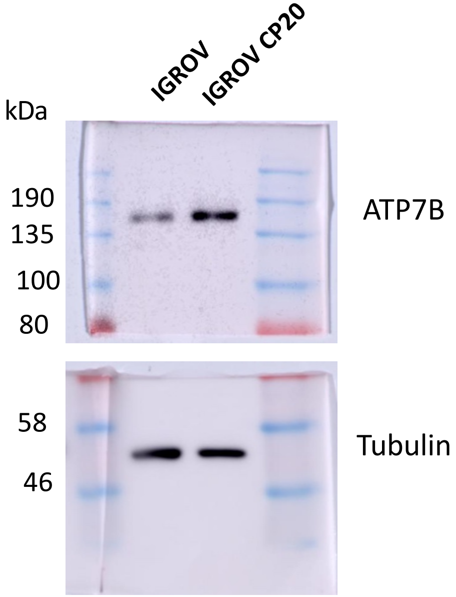

Original Western blots for Figure 1F

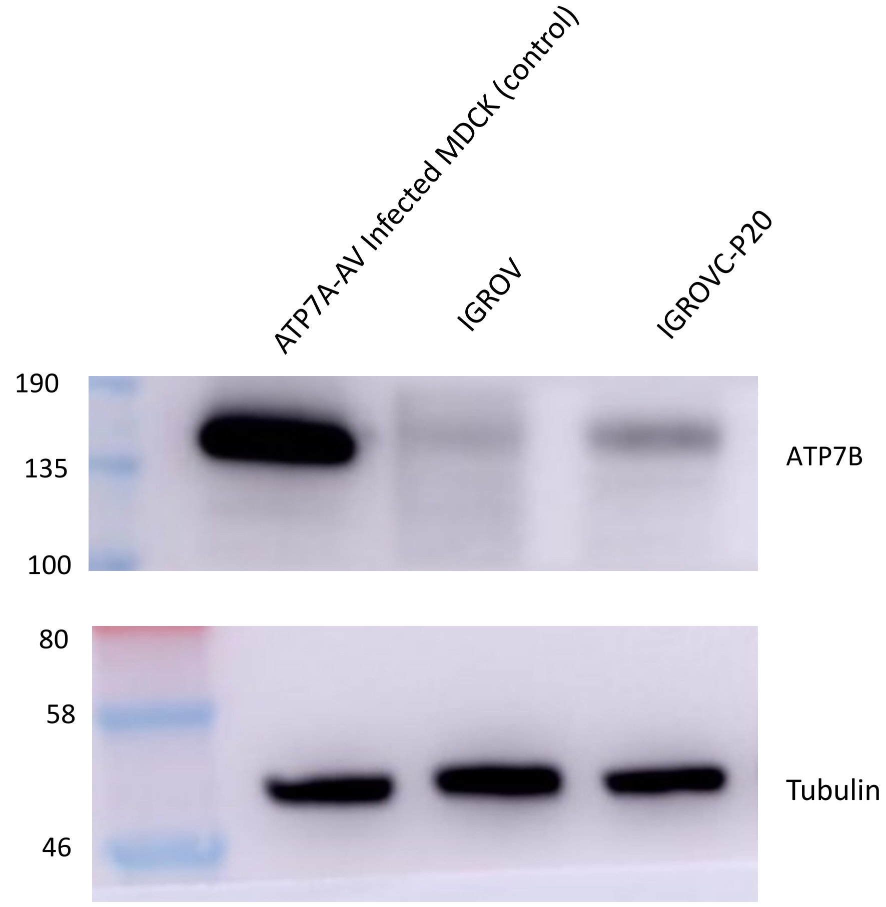

Original Western blots for Figure 1I

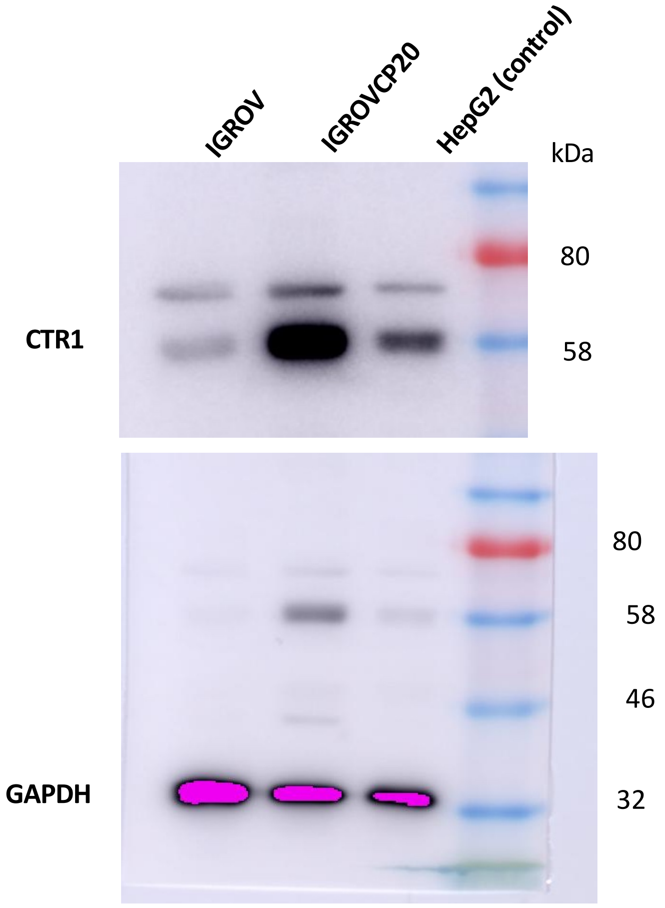

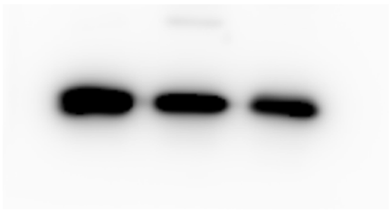

GAPDH lower exposure

Original Western blots for Figure 4B

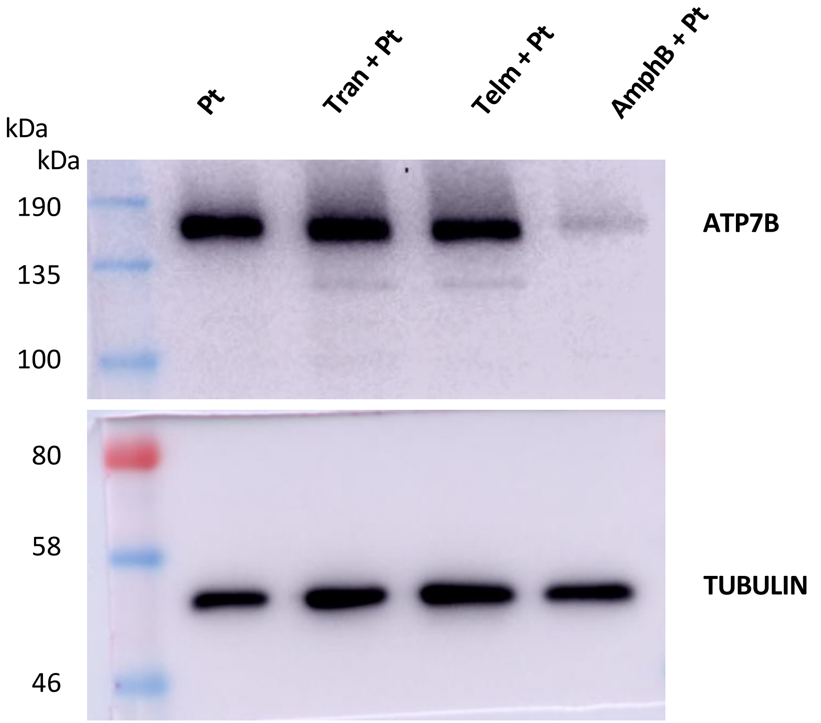

Original Western blots for Figure 4D

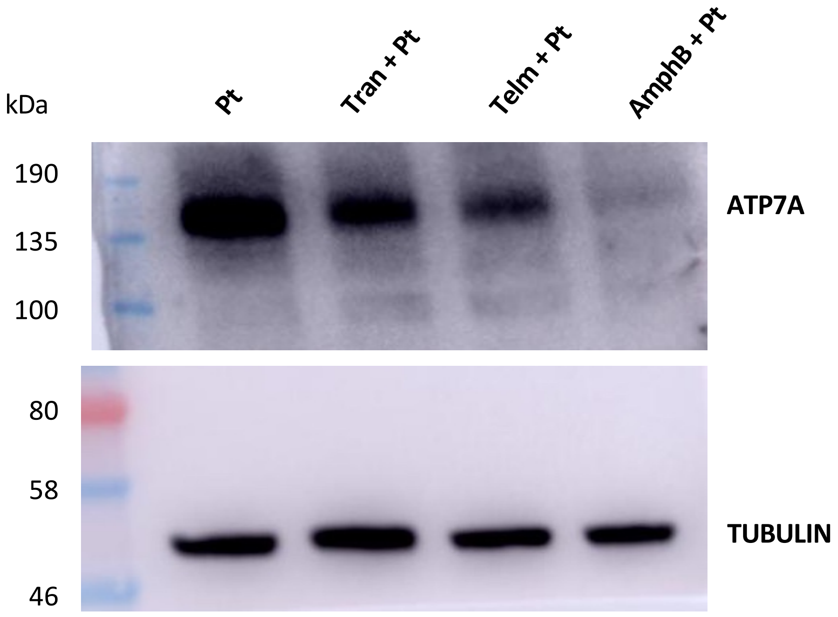

Original Western blots for Figure 4F

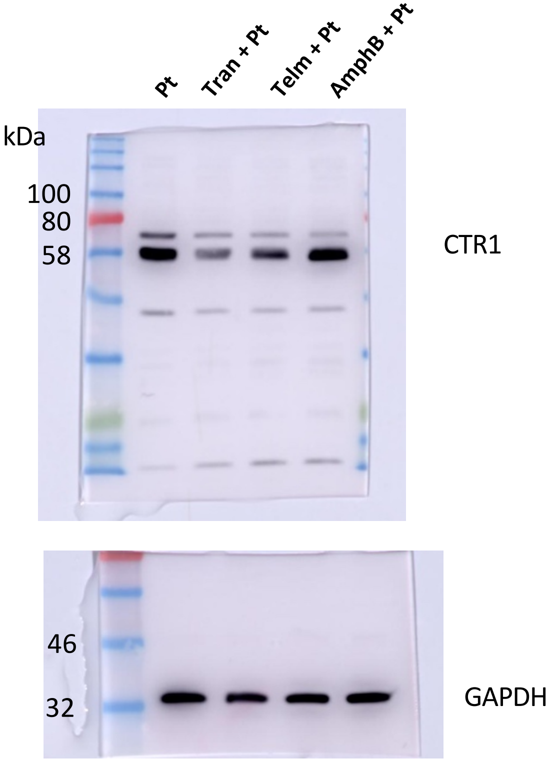

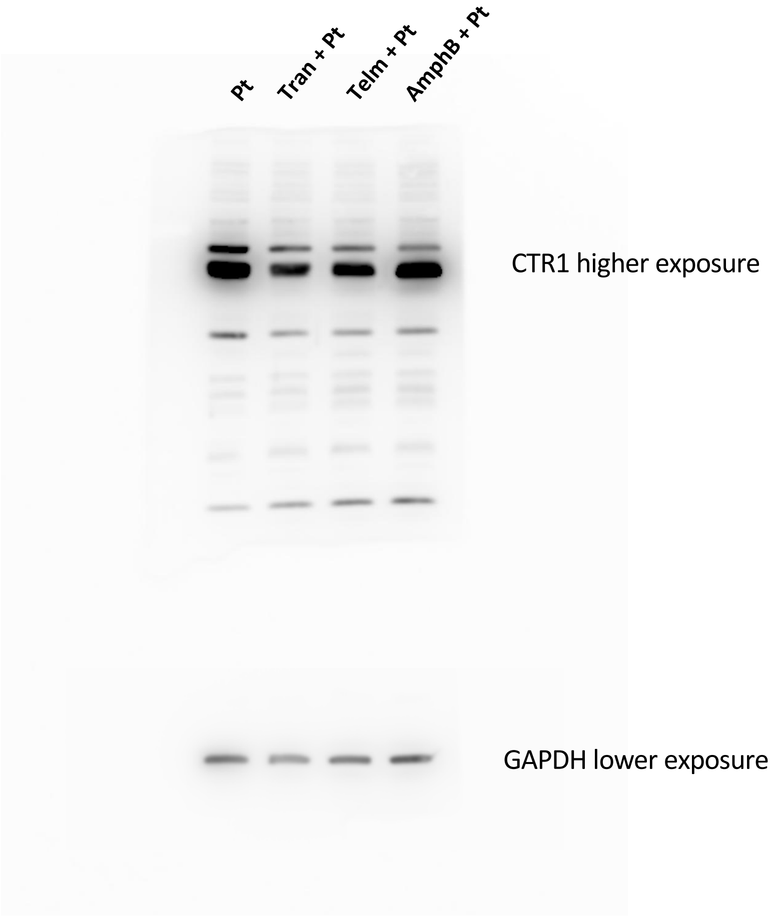

Supplement: Supplementary file 1 [file cancers-12-00608-s001.zip › Full Western blot images.pdf]
